# Wright was right: leveraging old data and new methods to illustrate the critical role of epistasis in genetics and evolution

**DOI:** 10.1093/evolut/qpae003

**Published:** 2024-01-18

**Authors:** Jorja Burch, Maximos Chin, Brian E Fontenot, Sabyasachi Mandal, Thomas D McKnight, Jeffery P Demuth, Heath Blackmon

**Affiliations:** Department of Biology, Texas A&M University, College Station, TX, United States; Department of Biology, Texas A&M University, College Station, TX, United States; Department of Biology, University of Texas at Arlington, Arlington, TX, United States; Department of Biology, Texas A&M University, College Station, TX, United States; Department of Biology, Texas A&M University, College Station, TX, United States; Department of Biology, University of Texas at Arlington, Arlington, TX, United States; Department of Biology, Texas A&M University, College Station, TX, United States; Interdisciplinary Program in Ecology and Evolutionary Biology, Texas A&M University, College Station, TX, United States

**Keywords:** epistasis, genetic architecture, line cross analysis, life history

## Abstract

Much of evolutionary theory is predicated on assumptions about the relative importance of simple additive versus complex epistatic genetic architectures. Previous work suggests traits strongly associated with fitness will lack additive genetic variation, whereas traits less strongly associated with fitness are expected to exhibit more additive genetic variation. We use a quantitative genetics method, line cross analysis, to infer genetic architectures that contribute to trait divergence. By parsing over 1,600 datasets by trait type, clade, and cross divergence, we estimated the relative importance of epistasis across the tree of life. In our comparison between life-history traits and morphological traits, we found greater epistatic contributions to life-history traits. Our comparison between plants and animals showed that animals have more epistatic contribution to trait divergence than plants. In our comparison of within-species versus between-species crosses, we found that only animals exhibit a greater epistatic contribution to trait divergence as divergence increases. While many scientists have argued that epistasis is ultimately of little importance, our results show that epistasis underlies much of trait divergence and must be accounted for in theory and practical applications like domestication, conservation breeding design, and understanding complex diseases.

## Introduction

Linking genotypes to phenotypes is a key challenge in biology. The mapping of genotype to phenotype is often simply called genetic architecture. However, genetic architecture has multiple components (e.g., identity of genes impacting a phenotype, structural arrangement of genes, and the mode of gene action). In this project, we focus on the mode of gene action, specifically, the degree to which divergence in phenotypes is due to additive, dominance, or epistatic modes of gene action.

Additive gene action occurs when the effect of an allele is independent of the genetic background. In contrast, dominance occurs when the effect of an allele is dependent on the other allele present at the same locus. Epistasis occurs when the impact of an allele is dependent on the genotype at other loci in the genome (Lynch & Walsh, 1998). Theoretical work demonstrates that additive gene action is the most effective substrate for adaptation (Lande & Arnold, 1983). When a trait is impacted by dominance, mutations that increase the fitness of an individual will take longer to fix than similar mutations with an additive architecture (Crnokrak & Roff, 1995). Finally, if the genetic architecture of a mutation is epistatic, the trajectory of evolution may be fundamentally different, and processes like population bottlenecks and population subdivision may be key in determining the rate and degree of adaptation (Cheverud & Routman, 1996; Goodnight, 1988; Wade & Goodnight, 1998).

Existing theory suggests that we should expect consistent differences in the type of genetic architecture associated with different types of traits. If a phenotype is strongly associated with the fitness of an organism (e.g., life-history traits), selection should quickly exhaust additive genetic variation for the phenotype as the most beneficial allele is fixed in the species (but see Laurie et al., 2004). These fitness-related traits should show primarily dominance and epistatic genetic variation. In contrast, traits less strongly associated with the fitness of an organism (e.g., morphological traits) may maintain additive genetic variation for a trait (Crnokrak & Roff, 1995; Roff & Emerson, 2006).

Widely accepted theories for phenotypes of crosses invoke different genetic architectures depending on the divergence of the included parental populations or species. For instance, when divergence is low, inbreeding depression is often ascribed to dominance effects (Lynch & Walsh, 1998). Dominance effects are also invoked to explain heterosis (Lynch, 1991). In contrast, when divergence is high, hybrid breakdown is often ascribed to epistasis leading to incompatibilities among lineages (Coyne et al., 1997; Orr, 2001). For example, in *Tribolium castaneum*, incompatibilities among diverged populations lead to postzygotic isolation, which is often caused by nonadditive genetic architectures (Demuth & Wade, 2007a). More broadly, between-species crosses are expected to show more complex architectures, especially epistatic interactions (Allen Orr, 2001; Coyne et al., 1997; Demuth & Wade, 2005; Lynch, 1991).

The number of plant and animal species known to hybridize is strikingly different. Approximately 25% of plant species but only 10% of animal species have been estimated to hybridize regularly (Mallet, 2005). Much of the theory underlying reproductive isolation is based on epistatic interactions among loci. An example of this is a Bateson–Dobzhansky–Muller incompatibility (BDMI), where two populations become incompatible when substitutions that are neutral in isolation have a deleterious fitness effect when present in the same individual. These recombinant genotypes have low fitness and limit the possibility of gene flow among populations or incipient species (Satokangas et al., 2020). One possible explanation for lower rates of hybridization in animals could be a result of more epistatic interactions in animal genomes relative to plant genomes. If animals exhibit more epistasis, they should also have a higher likelihood of developing BDMIs (limiting opportunities for hybridization) in animals.

Debate over the relative importance of epistatic versus additive gene action began during the modern synthesis. R.A. Fisher believed that migration was a sufficiently powerful homogenizer of genetic variation. Furthermore, he believed that most species could be modeled as panmictic with little substantive difference in genetic background and, consequently, that adaptation would proceed primarily through fixing mutations based on their additive effects (Fisher, 1958). In contrast, Sewall Wright viewed species as more loosely held together collections of populations where epistatic variation could have an important role in adaptation because interactions among loci within each deme’s local genetic background could influence the trajectory of evolution (Wright, 1931). Despite nearly a century of research, this debate has continued without a clear resolution (Ågren, 2021; Coyne et al., 1997, 2000; Peck et al., 1998; Stern et al., 2022; Wade & Goodnight, 1998). The goal of this investigation is to contribute to this discussion by documenting the proportion of trait divergence due to epistatic variation across hundreds of datasets spanning both plants and animals.

One reason that a clear consensus has not been reached is that not all methods can effectively detect epistatic variation. For instance, traditional GWAS, QTL, and variance partitioning methods have relatively limited ability to detect epistatic effects (Demuth & Wade, 2006). A second reason for the continued debate is that line cross analysis (LCA), one of the best methods, has only been used by a handful of evolutionary biologists (Demuth & Wade, 2007a; Demuth et al., 2014; Edmands et al., 2009; Fox et al., 2009; Miller et al., 2003). Additionally, past syntheses of available data have been limited in the number of studies (49) that they collected for synthesis (Roff & Emerson, 2006). Finally, studies that have used LCA to investigate genetic architecture have largely ignored model selection uncertainty leading to the possibility that existing estimates are biased by inherent problems with model selection (Blackmon & Demuth, 2016).

We address these limitations of past work by collecting 1,580 datasets from the literature, generating four datasets in *Solanum*, and incorporating 22 unpublished datasets from *Anaxyrus* hybrids. We incorporated these datasets into a public database (evobir.shinyapps.io/lca-synth/) where users can explore the results of applying a consistent analysis approach across 1,606 datasets and download all original data. We show that traits tightly associated with fitness typically have little additive variation and that life-history traits show more epistatic genetic effects than morphological traits. We also demonstrate that epistasis contributes more to trait divergence in animals than in plants and that animals (but not plants) tend to have more complex genetic architectures as divergence increases.

## Methods

### Solanum crossing data

**Figure 1.**
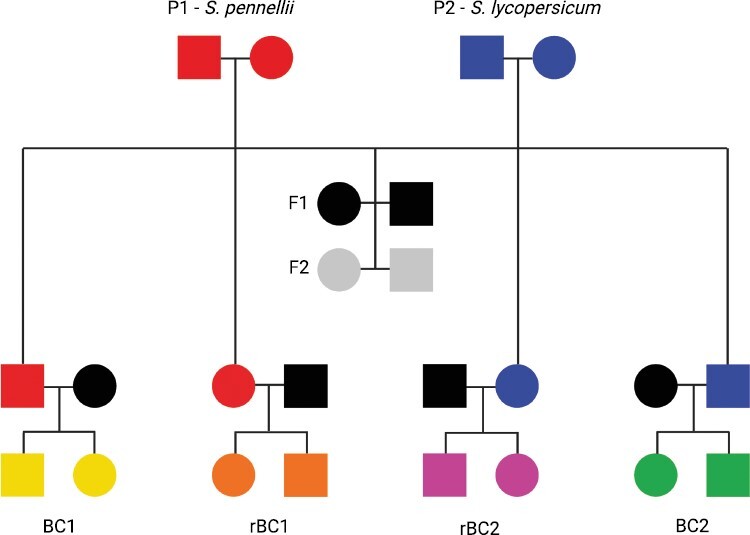
*Solanum* pedigree. Pedigree describing the cross design of the seven cohorts involving *S. pennellii* and *S. lycopersicum*. Note that black circles and squares on the second row from the bottom are F1 individuals used to generate back crosses. These are not connected to other parents to simplify the pedigree diagram.

Seeds for *Solanum lycopersicum* (cultivar VF36), *S. pennellii* (accession 716), and F1 hybrids (accession LA4135) were obtained from the C.M. Rick Tomato Genetics Resource Center at the University of California, Davis. These plants were used to generate seven cohorts (e.g., P1, F1, BC1, etc.) for inclusion in this study (Table 1; Figure 1). Seeds were treated with a solution of 20% commercial bleach for 20 minutes and then germinated on soil. Seedlings were grown to germination in a growth chamber (23 °C, 12-h photoperiod, 50% humidity), then transplanted and relocated at eight weeks to a greenhouse. The temperature in the greenhouse was maintained at approximately 23 °C, while humidity and lighting conditions were allowed to vary. This study was carried out at the experimental greenhouse of the Biology Department of Texas A&M University, which is located at 30.617 N and 96.355 W. For each of the seven cohorts, data was collected for germination time, time to anthesis, chlorophyll content, and above-ground biomass.

**Table 1.** Description of cohorts. The F1 family is listed to document its genetic makeup but is not included in the study. Strain numbers are those used by the UC Davis C.M. Rick Tomato Genetics Resource Center. The description is written in sire by dam format.

Family	Description of cross
P1	*S. pennellii*—LA0716 desert parent (self-pollinated)
P2	*S. lycopersicum—*VF36 domestic parent (self-pollinated)
F1	*S. pennellii *× *S. lycopersicum* (LA4135)
F2	F1 (self-pollinated)
BC1	*S. pennellii* × F1
rBC1	F1 × *S. pennellii*
BC2	*S. lycopersicum* × F1
rBC2	F1 × *S. lycopersicum*

### *Anaxyrus* crossing data

We examined 141 laboratory created hybrids between *Anaxyrus* (toads, formerly *Bufo*) species (Table 2), measuring the following morphological characters (following the methods of (Smith & Duellman, 1971); as modified by (Mendelson, 1994)): snout-vent length, tibia length, foot length, head width, head length, tympanum diameter, diameter of the orbit (internal distance between bony margins of orbit), posterior crest width (distance between lateral edges of supraorbital crest, taken from anterior side of junction with preorbital and canthal crests), anterior crest width (distance between lateral edges of supraorbital crest, taken from posterior side of junction with preorbital and canthal crests), eye-nostril distance (distance from posterior edge of bony margin of orbit to posterior margin of nostril), supratympanic spur length (straight distance between anterior and posterior margins of supratympanic crest, including some measure of the thickness of the postorbital crest), finger one length (distance from medial edge of pollical tubercle to tip of digit), finger three length (distance from base between second and third fingers to tip of digit), parotoid gland length (straight distance from most anterior point to most posterior point of gland), and parotoid gland width (straight distance across parotoid gland, taken near the midpoint along its length and oriented perpendicular to the longitudinal axis of the gland).

**Table 2.** Description of *Anaxyrus* crosses. Preserved specimens of hybrid offspring from a subset of laboratory crosses performed by W.F. Blair (1972) were examined to evaluate morphological variation in hybrids. This description is written in sire by dam format. T, W, and H abbreviations indicate *A. terrestris*, *A. woodhousii*, and *A. hemiophrys*, respectively.

	Species crosses
P1	*Anaxyrus terrestris* (T)
P2	*Anaxyrus woodhousii* (W)
P3	*Anaxyrus hemiophrys* (H)
F1	*A. terrestris *× *A. woodhousii* (T × W)
F1	*A. woodhousii *× *A. terrestris* (W × T)
F1	*A. terrestris *× *A. hemiophrys* (T × H)
F1	*A. hemiophrys *× *A. terrestris* (H × T)
F2	(T × W) × (T × W)
F2	(T × H) × (T × H)
BC1	(T × W) × T
rBC1	T × (T × W)
BC2	(T × W) × W
rBC2	W × (T × W)
BC3	H × (T × H)
rBC3	(T × H) × T
BC4	(H × T) × H

Adult specimens of both sexes were included, and juveniles were excluded from the analyses to control for ontogenetic variation. All specimens were fixed in 10% buffered formalin and preserved in 70% ethanol for at least 1 year prior to measurement to account for shrinkage artifacts resulting from the preservation process (Hayek et al., 2001; Lee, 1982). All measurements were taken using digital calipers to the nearest 0.01 mm.

### Literature search

To incorporate all possible data, we conducted an extensive literature search. Google Scholar was used to search the phrases “line cross analysis,” “analysis of means,” and “genetic architecture.” Additional records were discovered by reviewing citations to key landmark papers (Blackmon & Demuth, 2016; Cavalli, 1952; Hayman, 1958; Mather & Jinks, 1971; Roff & Emerson, 2006). This process led to hundreds of papers that reported results from line crosses. However, many of these could not be included because only estimated architectures rather than underlying phenotype measures were reported. In some cases, phenotypes were only reported in plots, not tables. In these cases, figures were enlarged, and a translucent grid was placed over the plot to estimate the original values. Some of the literature did not include information such as standard errors for line means or the directionality of crosses. To overcome these issues, we tracked and evaluated the impact of datasets that (a) failed to report uncertainty measures for the line means or (b) failed to identify the direction of crosses (lacked sex information for the parents). Datasets were included that contained at least one second-generation cross (e.g., F2 or backcross).

### Trait partitioning

To understand how genetic architecture varies when traits are closely associated with fitness versus when traits are loosely associated with fitness, we partitioned traits into two categories: life history and morphological. Obviously, not all traits fit into one of these two categories and researchers may disagree on the categorization of some traits. Some of the traits that are included in our study have been examined previously (Roff & Emerson, 2006). For these traits, we maintained past categorizations. We partitioned traits not included in previous studies based on expected association with fitness. Traits closely related to the organisms’ fitness were categorized as life history, while traits less associated with fitness were categorized as morphological. Within our morphological category, we included a number of traits that were not morphological or life history but that we predict to have little association with fitness (e.g., oil concentration, glycoalkaloid content, etc.) (Supplementary Table 1). We recognize that this categorization is one possible area that could introduce subjective judgment in our study. To determine whether our categorization produced two trait groups that likely differ in fitness association, we conducted a survey that allowed participants to score traits for their association with an organism’s fitness. Respondents were asked to score each trait from one (least associated with fitness) to ten (most associated with fitness). Responses were collected from 34 students and faculty from biology departments. These survey results provide an independent measure as to whether our categorization of traits into life history and morphology effectively parsed traits into those with high and low associations with the fitness of an organism.

### Line cross analysis

LCA is a quantitative genetics approach that uses phenotypes that are measured in the offspring of multiple crosses (e.g., P1, P2, F1, BC1, etc.) made between two different parental populations, strains, or species. These offspring are referred to as cohorts. Older methods of LCA used the joint-scaling test, a forward variable selection weighted least squares regression. This approach would fit a simple additive model and then apply higher-order composite genetic effects until a likelihood ratio test indicated no significant improvement to the model (Mather & Jinks, 1982). This forward model selection method of joint-scaling is well documented to lead to inconsistent “best” models and biased parameter estimates (Derksen & Keselman, 1992). Additionally, this approach fails to account for uncertainty in model selection (Burnham & Anderson, 2002). We used a full information-theoretic approach to LCA to alleviate these issues allowing us to generate model-averaged parameter estimates (Blackmon & Demuth, 2016).

LCA was used to infer the composite genetic effects (i.e., autosomal additive, maternal additive, autosomal additive by additive, etc.) that contribute to trait divergence for all datasets. Many types of genetic effects are possible, and what those are depends on the crosses included in a particular dataset. These effects can be defined both by the portion of the genome they reside in: autosomal, cytotype, maternal, or sex chromosomes as well as the mode of action (additive, epistatic, dominance). LCA uses a mean and standard error for each cohort included in a study. Since each line in a study will have different proportions of the two parental genomes and different levels of heterozygosity, the phenotype for each line has different opportunities to be impacted by different composite genetic effects. This characteristic allows us to construct a matrix of coefficients (Supplementary Table 2) that describes the opportunity for each possible genetic effect to impact trait divergence in each experimental dataset (Lynch & Walsh, 1998). Different composite genetic effects can be evaluated based on the crosses that an experiment uses.

LCA is a weighted least squares model that allows us to represent the genetic architecture as a linear model (1)


$$\begin{array}{l} y=C\beta \;+\;e \end{array}$$ (1)

where y represents the vector of observed line means, C is the C-matrix that describes the opportunity for each genetic effect to impact the phenotype of a line, β is the vector of parameters to be estimated that describe the degree to which each composite genetic effect is responsible for the observed line means, and e is a vector of the random errors associated with the means of each cohort. In the weighted least squares approach, we then find the estimate of the parameters $\hat{\beta }$ that minimize the weighted sum of squares (2).


$$\begin{array}{l} {\left(y-C\beta \right)}^{T}V^{-1}(y-C\beta ) \end{array}$$ (2)

here V is the variance–covariance matrix of e. In LCA, V is a diagonal matrix with the squared standard errors of cohort means along the diagonal. This scales each cohort’s contribution to the sum of squares by the certainty of the cohort mean. This means that if there is large uncertainty in a mean for a given line, it will contribute less to the sum of squares and, by extension, to the inference of the genetic architecture for the trait.

We used the software SAGA 2.0 to evaluate all possible models for the genetic architecture of our traits with the exceptions described below that reduce the size of model space for a handful of datasets (Armstrong et al., 2019). In the analyses of each dataset, we limited the space of possible models to be fit to those with one fewer parameter (composite genetic effects) than the number of cohorts included in the experiment, but we set a maximum number of parameters to seven. Limiting the maximum number of parameters to seven is necessary to avoid model spaces that are computationally intractable for the handful of datasets with the largest number of cohorts. Using this approach led to a model space ranging from 5 to 204,733 models of genetic architecture. A proportion of these models will include genetic effects where the coefficients in the C-matrix are highly correlated leading to difficulty in calculating the maximum likelihood estimate of parameters. Models that exhibit this characteristic are dropped from the analysis, and parameters are estimated by the remaining models. Previous simulation studies indicate that this does not lead to significant bias or loss of power in the inference of composite genetic effects under a model-averaging approach (Armstrong et al., 2019; Blackmon & Demuth, 2016). The AICc (small sample size corrected version of AIC) score for each evaluated model was recorded, and we constructed a 95% confidence set of models that were used to produce model-averaged results that account for model selection uncertainty (Burnham & Anderson, 2002). Datasets where the parental sex was unknown were analyzed under a simplified model set. This simplified model set included additive and dominance genetic effects, and three epistatic interactions: additive by additive, additive by dominance, and dominance by dominance. Model averaging can be done in several fashions. The approach implemented in the R package SAGA calculates a model-averaged genetic effect based on Akaike weights (3)


$$\begin{array}{l} \hat{\theta }=\overset{R}{\underset{i=1}{\sum }}w_{i}\cdot \;\hat{\theta_{i}} \end{array}$$

where $\hat{\theta }$ is the model-averaged parameter estimate calculated by summing across all models that include the current parameter multiplying the parameter estimate from each model ($\hat{\theta_{i}}$) by that model Akaike weight ($w_{i}$). With this approach, models that lack a parameter of interest effectively provide evidence that that parameter is zero.

### Statistical analysis of LCA results

For each dataset, we recorded the genetic effects that had a variable importance greater than 0.5 and a 95% confidence interval that excluded zero (Blackmon & Demuth, 2016). Variable importance describes the summed Akaike weight of all models with a particular genetic effect. Previous simulation studies suggest a variable importance cutoff of 0.5 is appropriate to maintain false positive rates below 5% (Blackmon & Demuth, 2016).

For this study, we chose to pool all genetic effects into classes of additive, dominance, or epistasis (described in Supplementary Table 4). However, genetic effects inferred from a dataset can take either positive or negative values (dependent on the choice of which parental line is chosen as the P1 versus P2). To avoid canceling out genetic effects, we took the absolute value of all significant effects and normalized by dividing each genetic effect by the sum of all significant effects. This allows all studies to be compared to one another on the same scale. By doing this, we produced results where each dataset (a phenotype in a particular cross) could have an epistatic contribution that varies from zero to one.

One potential challenge in our study is that some authors have generated many datasets for the same species and trait pairings. For instance, in crosses of *Zea spp.*, Srinivasan & Brewbaker (1999) produced 28 crosses of the same two species using different domesticated lines as one of the parents and measured plant height in all cohorts produced. In contrast, other authors investigating plant height have generated only single datasets (Khodambashi et al., 2012; Macnair & Cumbes, 1989). To avoid allowing this variation in representation among species and phenotypes to dominate results, we first performed a consolidation step. We averaged across results from datasets that shared a species and phenotype. For instance, in *Tribolium castaneum* we had 27 datasets that investigated the number of offspring produced in crosses among various populations (Demuth, 2004). For downstream analyses, these were combined by calculating the mean additive, dominance, and epistatic contributions across all 27 datasets.

To assess significance when comparing different data types, we used a permutation test to estimate empirical *p*-values. For instance, to evaluate whether life-history and morphological traits have different epistatic contributions, we first calculated the observed mean difference by computing the difference in the mean epistatic contributions for all life-history datasets versus all morphological datasets. Next, we shuffled the assignment of datasets into life history or morphological and calculated a simulated mean difference. We repeated this process of calculating simulated differences 10,000 times to generate a null distribution to which we compared the observed mean difference.

Scripts for all analyses are available in a GitHub repository: https://github.com/coleoguy/LCAdata.

## Results

### Development of online database

Our literature search and original work yielded 1,606 datasets. These records were previously scattered among 168 articles, books, and theses, often behind paywalls, or the underlying data was only available in a graphical format. We used the R package Shiny version 1.7.1 to create a freely available, interactive database that houses all 1,606 datasets analyzed in this study (https://evobir.shinyapps.io/lca-synth/). The database generates a plot of epistatic contribution to phenotypes in all crosses. However, these epistatic contributions can be parsed into six different comparisons: clade (plant vs. animal), divergence (within vs. between species), trait class (life history versus morphological), organism type (domestic vs. lab vs. wild), sex chromosome system (none vs. XY vs. ZW), and regression type (weighted versus unweighted). The data used to generate the plot can also be subsetted (i.e., using only within-species crosses) using the provided drop-downs. Users may also select the “Table” tab to view a comprehensive reference list of all datasets analyzed. The last column of the table includes links to download each original dataset. The final tab includes a list of citations for all datasets.

### Trait partitioning

Of the 444 unique traits, 220 were categorized as life history and 224 as morphological. From the survey results, the mean fitness-association value for traits (ranked from 1 to 10) categorized as life history was 6.90 and 4.78 for morphological, these differences were significant based on a *t*-test (*p*-value < .001) (Supplementary File 1). This result aligns with our categorization given that life-history traits are more associated with fitness than morphological traits.

### Primary analyses

Of the 1,606 datasets, 294 were analyzed under standard LCA conditions, and 1,312 datasets were analyzed under a simplified model set, because the original studies failed to report the sex of parents. Another challenge was the presence of 620 datasets where the authors failed to report uncertainty in measured phenotypes (note that studies may fall into more than one of the categories described). Below we describe the analyses we performed to assess the impact of these various data deficiencies. One of the main justifications for the information-theoretic approach that we employ is the incorporation of model selection uncertainty into parameter estimates. With this approach, we only include datasets in downstream analyses if one or more composite genetic effects have confidence intervals that do not overlap zero and a variable importance greater than or equal to 0.5. Of the 1,606 analyzed datasets, 81% (1,297) met these thresholds. Across all analyzed datasets, the number of models included in the confidence model set ranged from 1 to 2002.

**Figure 2.**
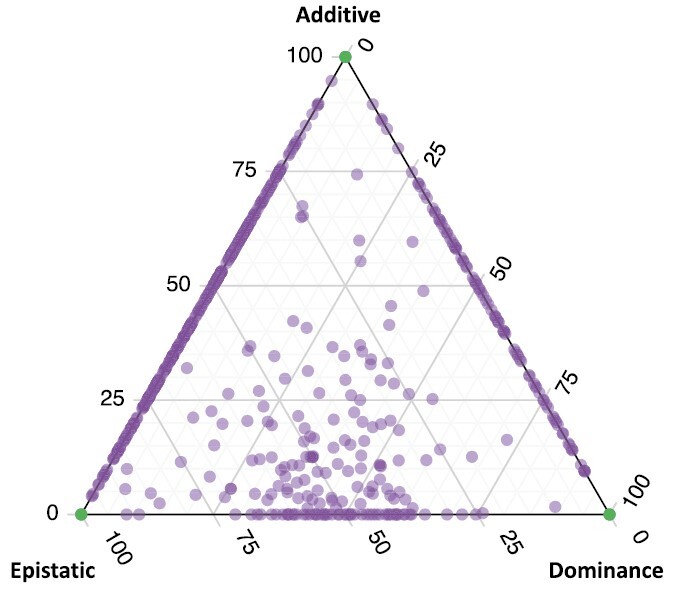
Composite genetic effects. The distance to a vertex in this plot describes the proportion of that particular genetic effect. For instance, points toward the top vertex have primarily additive effects, while those near the left are primarily epistatic. Each analyzed dataset is plotted as a single point which describes the degree of contribution from additive, dominance, and epistatic modes of gene action. Many points overlap in some regions, and the darkness of points indicates the relative number of overlapping points.

After completing LCA, we pooled composite genetic effects into additive, dominance, and epistatic effects and scaled these three values to sum to one. We found that 763 datasets had contributions of only one class of genetic architecture. Of these 763 datasets, 528 were purely additive (green point at the top vertex of Figure 2), 187 were purely epistatic (green point at the lower left vertex of Figure 2), and 48 were purely dominance (green point at the lower right vertex of Figure 2). The 259 datasets that fall on the line between additive and epistatic have no dominance component (Figure 2, left side). The 89 datasets that fall on the line between additive and dominance have no epistatic component (Figure 2, right side). Finally, the 105 datasets that fall on the line between epistatic and dominance have no additive component (bottom of Figure 2). The remaining 81 datasets have significant contributions from all three genetic categories and fall within the plotting field.

For downstream analyses, we combined results that originated for each unique species-trait pairing. This process led to 488 results, which are discussed below. To investigate the relative importance of epistasis in trait divergence, we analyzed the differences in epistatic contributions between the groups shown in Table 3.

**Table 3.** Comparisons for permutation tests. We use the term morphological for convenience. This category includes data for a variety of phenotypes that we judge (or historically have been judged) not to be tightly associated with fitness.

Group 1 (*N*)	Group 2 (*N*)
Life history (244)	Morphological (244)
Within-species (397)	Between-species (91)
Plants (362)	Animal (126)

**Figure 3.**
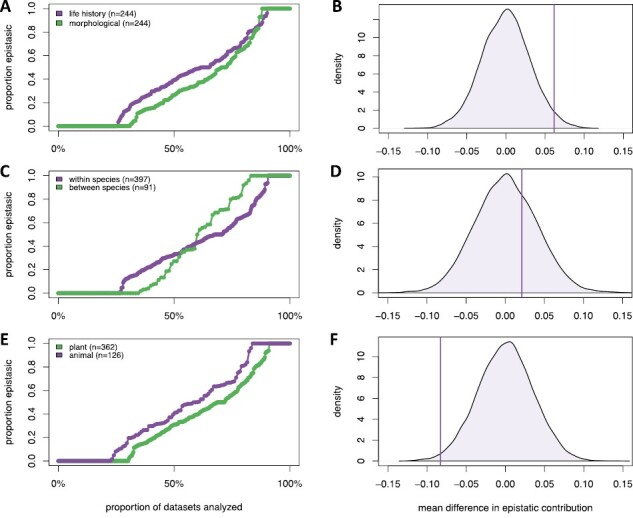
Epistatic contribution to trait divergence. Results from each dataset are sorted by epistatic contribution and then plotted across the horizontal axis based on the total number of datasets. The vertical axis indicates the epistatic contribution of a given dataset. The plots on the right represent the result from a permutation test for each analyzed comparison. (A, B) Comparison of life-history versus morphological traits. (C, D) Comparison of within-species versus between-species crosses. (E, F) Comparison of plant versus animal species.

Life-history traits showed a greater proportion of epistatic contribution to trait divergence than morphological traits (mean difference of 0.06, empirical *p*-value .024; Figure 3A and B). For the morphological traits (*N* = 244), 30 datasets had an epistatic contribution of one, and 76 datasets had an epistatic contribution of zero. For the life-history traits (*N* = 244), 24 datasets had an epistatic contribution of one, and 63 datasets had an epistatic contribution of zero. In a comparison of the proportion of dominance effects within genetic architecture, we did not find a significant difference between life-history and morphological traits (*p*-value > .9).

When comparing epistatic contribution to trait divergence in within-species versus between-species crosses, we did not find a significant difference in epistatic contribution (mean difference of 0.03, empirical *p*-value = .29, Figure 3C and D). For the within-species crosses (*N* = 397), 38 datasets had an epistatic contribution of one, and 107 datasets had an epistatic contribution of zero. For the between-species crosses (*N* = 91), 16 datasets had an epistatic contribution of one, and 32 datasets had an epistatic contribution of zero.

In our comparison between plants and animals, we found a significantly higher epistatic contribution to trait divergence in animals than plants (mean difference of −0.08, empirical *p*-value = .01; Figure 3E and F). For plants (*N* = 362), 33 datasets had an epistatic contribution of one, and 110 datasets had an epistatic contribution of zero. For the animals (*N* = 126), 21 datasets had an epistatic contribution of one, and 29 datasets had an epistatic contribution of zero.

To further examine the difference in epistatic contribution to trait divergence between plants and animals, we compared within- and between-species crosses in only animals and then repeated the comparison with only plants. In the comparison including only animal datasets, we found a significantly higher epistatic contribution in the between-species crosses (mean difference of 0.3, *p*-value = .0014). However, for the crosses in plants, we did not see a significant difference in epistatic contribution between the within- and between-species crosses (mean difference of −0.04, *p*-value = .79).

### Secondary analyses

**Figure 4.**
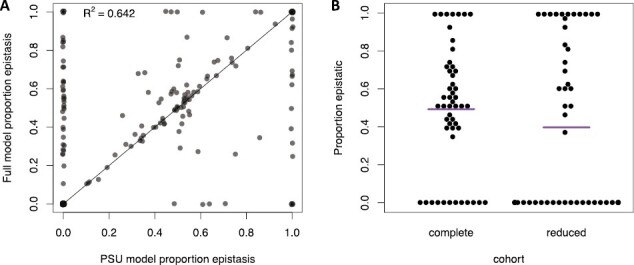
Impact of variable data. (A) The vertical axis indicates the epistatic contribution to trait divergence when datasets are run against the maximal model set, while the horizontal axis indicates the epistatic contribution of the same datasets run against a reduced model set consisting of just five composite genetic effects. (B) Epistatic contribution of datasets with 16 cohorts (complete) versus the same datasets reduced to a 5-cohort set (reduced). The purple line indicates the arithmetic mean.

To ensure that the inclusion of datasets where authors failed to report parental sex did not bias our results toward more or less detection of epistatic effects, we completed a separate test on the datasets where parent sex was specified (*N* = 294). We first analyzed these datasets using the full model set (made possible by parent sex information) and then reran each dataset as though parent sex was unavailable, which limits the possible composite genetic effects to additive, dominance, additive by additive, additive by dominance, and dominance by dominance. This experiment revealed that for 57% of datasets, analysis without parental sex information led to no change in the estimated epistatic contribution to trait divergence (Figure 4A). However, we did observe a slight bias toward detecting more additive effects when parental sex was withheld (29% of datasets). Finally, the remaining 14% of datasets showed an increase in the estimated epistatic contribution in the absence of parental sex information. We used a *t*-test to determine if the proportion of epistatic genetic effects were different between datasets with or without sex included and found a *p*-value of .51. Based on these results, we included datasets where parental sex was absent in all primary analyses.

A portion of the datasets have a large number of cohorts (16), whereas many datasets have fewer cohorts. We completed an additional test to determine if datasets with fewer cohorts were biased to detect more or less epistasis than those with many cohorts. Forty datasets were selected that included the same set of 16 cohorts; these forty datasets were all generated using *Tribolium castaneum* and focused on phenotypes associated with reproductive isolation (Demuth & Wade, 2007a,b). These files were first analyzed through LCA under standard conditions. Then these same datasets were reduced to a subset of the original 16 cohorts to only include five cohorts: P1, P2, F1, BC1, and BC2 (Supplementary Table 3). These thinned datasets were subjected to LCA in a standard fashion. We then compared the epistatic contributions of both the complete-cohort analysis and the reduced-cohort analysis and found that datasets with a reduced number of cohorts detect less epistatic effects than those with a larger number of cohorts (Figure 4B).

Thirty-one of these 40 datasets had intermediate epistatic effects (e.g., an epistatic component between zero and one) in the complete-cohort analysis. When these datasets were subset to a smaller number of cohorts, 17 of the datasets with an intermediate epistatic effect shifted to have either no epistatic effects (13 datasets) or epistatic effects only (four datasets). This slight bias toward the detection of less epistatic effects was not severe. We used a *t*-test to determine whether the proportion of epistatic genetic effects were different and found a *p*-value of .65, suggesting that the differences in cohort size have limited systematic impacts on our inference. Furthermore, in some sense, a positive result in our study is detecting epistatic contributions, so we have learned that LCA is a conservative approach biased toward detecting only additive effects when data is sparse. Based on these findings, we retained all datasets in our study, regardless of the number of cohorts included in the original study.

## Discussion

Previous studies have shown the importance of epistatic genetic effects in the genetic architectures underlying trait divergence under various conditions and in certain species (Lynch, 1991; Roff & Emerson, 2006). Unlike previous studies, we did not use results from independent studies that employed variable methods and heuristics to infer genetic architecture. Instead, we have used a unified and consistent approach to quantify the proportion of trait divergence due to epistatic genetic effects across the tree of life, resulting in the largest study of its type (total sample size of 1,606 and taxonomic breadth of 65 genera).

In our analysis of trait type, we included only two categories of traits: life history and morphological. Some previous studies have included four categories of traits (life history, morphological, physiology, and behavior). A study by Crnokrak and Roff (1995) found that, as predicted, life-history traits exhibit a greater epistatic contribution than morphological traits. Though we did not include separate categories for behavioral and physiological traits, through our analysis of over 1,600 datasets, we have shown results that align with Cnrokrak and Roff’s findings, where traits more associated with fitness show a greater contribution of epistatic genetic effects than traits less associated with fitness. This result is particularly interesting when we consider that traits that are key to fitness have epistatic architectures that can be converted to additive effects in the event of a population bottleneck (Barton & Turelli, 2004; Cheverud & Routman, 1996; Cheverud et al., 1999; Goodnight, 1988; Hill, 2017). We use the term “converted” but recognize that the total epistatic variation is not directly converted to additive, rather it is a convenient word that describes the source of the new additive variation (Barton & Turelli, 2004; Hill, 2017). This bottleneck might be caused by a mismatch between phenotype and a recent shift in the adaptive landscape. Thus, epistatic variation could function as a capacitor for adaptive variation that is converted to additive variation during a bottleneck event. This additive variation can then be selected to improve the match between the organism’s phenotype and its environment—a rarely considered form of preadaptation.

While we highlight the role of epistasis in the genetic architecture of trait divergence. We recognize that LCA could infer epistatic effects that did not exist at any point in the history of the populations being studied. For instance, mutations could occur during the divergence of strains that then generate novel multilocus genotypes in the cohorts produced during LCA (not dissimilar to a classic model of BDMIs; Satokangas et al., 2020). However, this seems unlikely if the parental strains are closely related. Furthermore, if this were a driving force in our results, we would expect to see a strong and consistent relationship between the importance of epistasis and the degree of divergence among strains (discussed below).

It has been suggested that as divergence increases, complex genetic architectures (containing epistatic effects) become more common (Lynch, 1991). In our results, we see this pattern only in animals (*N* = 268) and not in plants (*N* = 1,238). To better understand this result, further analysis should be done to look at specific divergence times and their relationship to epistatic contribution. We completed this analysis but were limited by the number of available divergence times between the crosses in our datasets (Supplementary Figure 1). We analyzed epistatic contributions as a function of divergence time for 44 of our datasets. Crosses, where divergence times were available, were split unequally between plants (27) and animals (17). To better understand the relationship between genetic architecture complexity and divergence time, more divergence times need to be documented for the organisms in our database.

Previously we have discussed our application of model selection uncertainty to reduce occurrences of bias due to ignored uncertainty in model selection. To do this, we omitted datasets from the analyses where all genetic effects had standard errors overlapping zero and/or did not have variable importance of 0.5 or greater. This criterion reduced our total number of datasets from 1,606 to 1,297, meaning that we were unable to estimate the genetic architectures for 309 datasets using our method. Many of these datasets were collected from previous publications that reported inferred genetic architectures. However, 19% of our datasets failed to meet these thresholds for inference of genetic architecture, highlighting the importance of assessing model selection uncertainty and the potential for false positives in more traditional approaches to LCA. We suggest that model averaging be included in future LCA studies to properly account for model selection uncertainty in parameter estimates. One possible alternative to this approach would be the development of a robust model adequacy framework applicable to LCA studies.

Another potential concern is the failure of most LCA studies to account for differences in ideal environments of parental cohorts. Unfortunately, the vast majority of studies do not assess whether the diverged cohorts have different ideal environments and instead are conducted in a single environment that is convenient for the researcher. This makes it impossible to investigate gene-by-environment (GxE) interactions that may well vary among cohorts and species. However, previous work that has extended LCA to include GxE interactions has shown in a handful of datasets that similar genetic effects are inferred when single or multiple environments are tested (Armstrong et al., 2019; Demuth & Wade, 2007b). Instead, the same basic genetic effects are inferred as well as additional GxE effects.

By analyzing this large amount of data, we can look at a trait’s association with fitness from a different perspective. Previously, we considered traits as either life history or morphological, and then determined the proportion of epistatic genetic effects underlying the trait divergence. Another way of determining how a trait may be related to fitness is to leverage our inference of genetic architecture. A trait table (Table 4) was constructed to identify the phenotypes with the highest proportion of epistatic contribution, presumably closely aligned with fitness, as well as the phenotypes with the lowest proportion of epistatic contribution, presumably loosely aligned with fitness. This table provides a unique perspective to determining how closely associated a particular trait is to the fitness of an organism. This can be accomplished by observing the proportion of trait divergence due to composite epistatic effects, and phenotypes with a higher epistatic contribution would be associated more closely with fitness than those with a lower epistatic contribution. We can make this assumption as we have shown that life-history traits (mating success, viability, fecundity) show more complex genetic architectures (containing epistatic effects) than morphological traits. The results of this analysis are dependent on the species of interest, as plant height, for example, could be more closely associated with fitness in some species than others.

**Table 4.** Mean epistatic composite genetic effects for phenotypes. Phenotypes with the highest and lowest mean epistatic composite genetic effect that included at least four datasets in our database. For phenotypes with a high epistatic effect, we selected all traits with a value of 0.75 or higher. For phenotypes with the lowest epistatic effect, we included all that had an effect of zero.

Phenotype	Frequency	Proportion epistatic
Total phenol content	4	0.93
Yield per plant	8	0.84
Viability with low food	4	0.75
Bundle strength	4	0.75
First siliqua height	4	0.75
Viability with high food/propionic acid	4	0.75
Chlorophyll concentration	6	0
Eggs (mites) per plant	4	0
Kernel rows per ear	17	0
Kernels per row	17	0
Body width	9	0
Wing size	6	0

By comparing large taxonomic groups (e.g., plant vs. animal), we are also effectively including the impact of many other traits that are different among these groups. For instance, plants exhibit striking diversity in mating systems, including many species capable of selfing. Self-fertilization is a form of inbreeding that occurs when an organism fertilizes its eggs using its own pollen or sperm rather than that of another individual. In contrast, animals rarely have the ability to self-fertilize. Our database includes one dataset from *Ophryotrocha puerilis*, a sexually and asexually reproducing polychaete that can potentially self-fertilize. One possible concern is that our inferences of differences in these large clades are due to these differences in the frequency of mating systems that allow for selfing. It is possible that when a species typically self-fertilizes, it will exhibit relatively low levels of genetic diversity, making it unlikely to exhibit epistatic variation dependent on having variation in the crossed populations, though it is possible that under moderate levels of inbreeding, selfing can lead to increased additive genetic variance. However, an alternative argument would be that the crosses used in these studies are specifically chosen because they have diverged phenotypes. Even if high levels of selfing have limited the opportunity for epistasis in any given population in the history of the species, when crosses are made between diverged strains, epistatic variation is likely to be revealed, not dissimilar to the way that migration can reveal epistatic variation (Wade & Goodnight, 1998). This observation would also be similar to the observation of hybrid dysgenesis due to BDMIs. A future analysis focusing on sister species where inbreeding has diverged dramatically would likely be the best way to reveal the potential for mating systems to drive inferences of genetic architecture.

With the vast amount of data analyzed, we could make comparisons between clades and cross-types. In comparing plants and animals, we found that animals have a greater contribution of epistatic effects to trait divergence than plants. This result is interesting as it is widely accepted that plants have higher rates of hybridization than animals (Mallet, 2005). Many factors can influence how successfully a species can hybridize, but a well-understood mode of reproductive isolation is BDMIs (a type of epistatic interaction). Whether the increased contribution of epistasis that we document in animals plays a causative role in broad patterns of hybridization should be investigated further.

## Supplementary material

Supplementary material is available online at *Evolution.*

qpae003_suppl_Supplementary_Materials

qpae003_suppl_Supplementary_Files

## Data Availability

Data and analysis scripts are available in a GitHub repository: https://github.com/coleoguy/LCAdata.
