# Supplementary material for: Wright was right: leveraging old data and new methods to illustrate the critical role of epistasis in genetics and evolution
Source: Evolution. 2024 Jan 18;78(4):624–34. doi: 10.1093/evolut/qpae003 (PMC10964199; doi:10.1093/evolut/qpae003)
Supplement: qpae003_suppl_Supplementary_Materials [file qpae003_suppl_supplementary_materials.pdf]

**Supplemental Table 1.** Condensed list of traits that were categorized as morphological or life history traits.

| Morphological Traits | Life History Traits |
|---|---|
| percent damage | % of eggs laid on mushrooms relative to tomatoes |
| 2.5% span length | 100-seed weight |
| locomotor activity | 20 year growth of survivors |
| 60 day bodyweight in females | 20 year survival |
| 60 day bodyweight in males | activity |
| abdominal length | age of 1st reproduction |
| abdominal width | average weight per fruit |
| above ground biomass | body weight |
| acetylene reduced | boll number |
| anthocyanin content | boll weight |
| aphid survival | fecundity |
| arachidic acid | fem develop time |
| ascorbic acid content | female starvation resist |
| beak length | male develop time |
| behenic acid | male starvation resist |
| biomass tolerance | condition factor |
| body depth | days to 50% flowering |
| body length | days to 50% silking |
| body shape | days to anthesis |
| body weight | days to first flowering |
| body width | days to first fruit set |
| branches per panicle | days to flower initiation |
| bristle size | days to harvest |
| bundle strength | days to heading |
| female weight | days to maturity |
| male weight | days to physiological maturity |
| calyx length | days to red fruit |
| canopy temp | days to ripe green fruit |
| canopy width | days to silking |
| chlorophyll content | development time |
| coleoptile length | difference in days between silking and pollen shedding |
| condensed tannins | dispersal tendency |
| corolla length | distance from stigma to anthers |
| cotyledon area | dry fodder yield |
| days taken to first lateral | early fecundity |
| diameter at breast height | egg dispersal |
| dry matter content | egg number |
| dry weight | egg size |
| ear diameter | egg survival |
| ear height | egg volume |
| ear length | eggs per plant |
| eggshell color | ejaculation |

| Morphological Traits | Life History Traits |
|---|---|
| eicosenoic acid | emergence |
| eye ball size | filled seeds/catkin |
| eye-nostril distance | first flowering |
| fatty acid concentration | fitness at high density of food |
| Fe root concentration | fitness at low density of food |
| Fe shoot concentration | fruit bearing index |
| finger 1 length | fruit diameter |
| finger 3 length | fruit length |
| first siliqua height | fruit size |
| flag-leaf angle | fruit weight |
| flag-leaf area | fruit weight per plant |
| flag-leaf duration | fruit weight per plot |
| flight time | fruit width |
| flower height | fruit yield |
| flower length | fruit-size index |
| flower width | fusarium rot resistance |
| fluctuating asymmetry | germination rate |
| foot length | germination success |
| fresh weight | grain weight |
| front femur length | grain yield |
| front femur width | grains per panicle |
| fruit shape | growth rate |
| genital length | growth rate length |
| gill raker length | growth rate mass |
| gill raker number | harvest index |
| ginning percentage | harvesting time |
| glycoalkaloid content | hatching success high salinity |
| grade index | hatching success low salinity |
| grain iron content | heading date |
| grain zinc content | herbivore density |
| gross viscosity | infection degree |
| head length | infection index |
| head width | intromission |
| herbivore density | kernel depth |
| hind femur length | kernel weight |
| internode length | kernels per spike |
| internode number | kernels per spikelet |
| iodine | keyhole limpet hemocyanin |
| K root concentration | latency period - disease |
| K shoot concentration | lifespan |
| kernel rows per ears | longevity, reared on bean |
| kernels per row | longevity, reared on chickpea |
| leaf area | maternal fitness |
| leaf discoloration red-green | mean stamen length |
| leaf length | mortality rate |

| Morphological Traits | Life History Traits |
|---|---|
| leaf length width ratio | mounting |
| leaf water potential | number of capsules |
| leaf weight | number of catkins |
| leaf width | number of days from seeding to pollen shedding |
| length of panicle | number of days from seeding to silking |
| lesion-diameter | number of days taken to flower |
| lesioned area | number of ears per plant |
| lesions per cm | number of eggs hatched |
| lesions per foliole | number of eggs per unit body weight |
| lesions per leaf | number of females |
| lignoceric acid | number of flowering stems |
| linoleic acid | number of flowers |
| lint percent | number of fruit |
| lint yield pp | number of fry to 10 months |
| long chain fatty acids | number of grains per spike |
| lower corolla lobe length | number of kernels |
| lower corolla lobe width | number of kernels per row |
| main stem length | number of locules |
| mesocotyl length | number of males |
| micronaire value | number of offspring |
| mid femur length | number of ovules |
| mineral matter | number of parthenocarpic fruits per plant of cumulative harvest |
| nicotine percent | number of parthenocarpic fruits per plant of first harvest |
| nitrogen fixation component | number of peduncles per plant |
| nornicotine percent | number of pods |
| number of adventitious roots | number of pods per plant |
| number of branches | number of racemes per plant |
| number of chaetigerous segments at sex change | number of ramets produced before the flowering season |
| number of clusters per plant | number of rows per ear |
| number of flower nodes | number of seeds |
| number of flowering stalks | number of seeds per fruit |
| number of lateral roots | number of seeds per plant |
| number of lesions | number of seeds per pod |
| number of leaves | number of siliquae per plant |
| number of mesocotyl roots | nursery survival at 6 months |
| number of mite gals | nuzzling |
| number of nodes | ovariole number |
| number of nodes above ear | ovary length |
| number of nodes below ear | panicles per plant |
| number of nodes per main stem | paternal fitness |
| number of primary branches | peak fecundity |
| number of primary laterals | peanut bud necrosis incidence percent |

| **Morphological Traits** | **Life History Traits** |
|---|---|
| number of red spots on the corolla | percent grains per panicle |
| number of secondary branches | percent of offspring deformed |
| number of secondary roots | percent survival |
| number of spike bearing culms | pericarp thickness |
| number of spikes per plant | pistil length |
| number of tillers per plant | |
| oil concentration | pod length |
| oleic acid | pod weight |
| orbit diameter | pod width |
| P root concentration | pod yield |
| P shoot concentration | proportion eggs on cowpea |
| palmitic acid | proportion of males |
| panicle length | proportion of survivors reproductive |
| parotid gland length | proportion that flowered |
| pedicel length | proportion that set fruit |
| peduncle length | proportion viable pollen |
| pelvic spine length | running speed |
| percent lesions | seed height |
| pericarp firmness | seed length |
| petiole length | seed per pod |
| photoperiod response to flowering date | seed pod ratio |
| plant height | seed set |
| plant size | seed size |
| plant spread | seed weight |
| plate number | seed width |
| pod length | seed yield |
| polyphenol oxidase activity | seed yield per plant |
| percent defoliation | seed yield pp |
| primary branch number | seeds per fruit |
| pronotum width | seeds per pod |
| ratio of cutting weight | sexual activity |
| reducing sugars | sperm length |
| residual variance in biomass tolerance | sperm receptacle length |
| root dry weight | straw yield per plant |
| root fractal | style length |
| root fresh weight | survival |
| root length | survival to eating high salinity |
| root weight | survival to eating low salinity |
| seed color | tassel length |
| seed oil content | time to find food |
| seedling height | time to first feeding |
| segment 7 length | time to germination |
| segment 8 length | viability |
| shelling percent | yield |
| shoot dry weight | yield per plant |

| Morphological Traits | Life History Traits |
|---|---|
| shoot fresh weight | yield per spike |
| shoot length | |
| shoot weight | |
| skin reflectance | |
| snout-vent length | |
| spike length | |
| spikelets per spike | |
| spine width | |
| stearic acid | |
| stem fractal | |
| stem weight | |
| sugar percent | |
| test weight | |
| thorax length | |
| thorax width | |
| tibia length | |
| total alkaloids percent | |
| total length | |
| total number of aphids | |
| total phenol content | |
| total soluble solids | |
| total soluble sugars | |
| tympanum diameter | |
| uniformity ratio | |
| wing area | |
| wing index score | |
| wing length | |
| wing size | |
| Zn root concentration | |
| Zn shoot concentration | |

**Supplemental Table 2 C-matrix.** Each row is representative of a different cross originating from the first two parental strains in row one and two. Each column represents a possible genetic architecture. Column names indicate the type of genetic architecture where capital letters are a portion of the genome (A: autosomal, X: X chromosome, Y: Y chromosome, C: cytotype) and lowercase letters represent types of architecture (a: additive, d: dominance) Mea and Med represent maternal effect additive and maternal effect dominance. For instance, YaAd represents an epistatic interaction between additive gene action on the Y chromosome and autosomal dominance.

| (sire x dam) | Aa | Ad | Xa | Xd | Ya | Ca | Mea | Med | AaAa | AaAd | AdAd | XaAa | XaAd | XdAa | XdAd | YaAa | YaAd | YaXa | CaAa | CaAd | CaXa | CaXd | CaYa |
|---|---|---|---|---|---|---|---|---|---|---|---|---|---|---|---|---|---|---|---|---|---|---|---|
| P1 | 1 | 0 | 1 | 0 | 0.5 | 1 | 1 | 0 | 1 | 0 | 0 | 1 | 0 | 0 | 0 | 0.5 | 0 | 0.5 | 1 | 0 | 1 | 0 | 0.5 |
| P2 | -1 | 0 | -1 | 0 | -0.5 | -1 | -1 | 0 | 1 | 0 | 0 | 1 | 0 | 0 | 0 | 0.5 | 0 | 0.5 | 1 | 0 | 1 | 0 | 0.5 |
| F1 | 0 | 1 | 0.5 | 0.5 | -0.5 | 1 | 1 | 0 | 0 | 0 | 1 | 0 | 0.5 | 0 | 0.5 | 0 | -0.5 | -0.25 | 0 | 1 | 0.5 | 0.5 | -0.5 |
| rF1 | 0 | 1 | -0.5 | 0.5 | 0.5 | -1 | -1 | 0 | 0 | 0 | 1 | 0 | -0.5 | 0 | 0.5 | 0 | 0.5 | -0.25 | 0 | -1 | 0.5 | -0.5 | -0.5 |
| F2a | 0 | 0.5 | 0.25 | 0.25 | -0.5 | 1 | 0 | 1 | 0 | 0 | 0.25 | 0 | 0.125 | 0 | 0.125 | 0 | -0.25 | -0.12 | 0 | 0.5 | 0.25 | 0.25 | -0.5 |
| F2b | 0 | 0.5 | -0.25 | 0.25 | 0.5 | 1 | 0 | 1 | 0 | 0 | 0.25 | 0 | -0.12 | 0 | 0.125 | 0 | 0.25 | -0.12 | 0 | 0.5 | -0.25 | 0.25 | 0.5 |
| rF2b | 0 | 0.5 | 0.25 | 0.25 | -0.5 | -1 | 0 | 1 | 0 | 0 | 0.25 | 0 | 0.125 | 0 | 0.125 | 0 | -0.25 | -0.12 | 0 | -0.5 | -0.25 | -0.25 | 0.5 |
| F2c | 0 | 0.5 | -0.25 | 0.25 | 0.5 | -1 | 0 | 1 | 0 | 0 | 0.25 | 0 | -0.12 | 0 | 0.125 | 0 | 0.25 | -0.12 | 0 | -0.5 | 0.25 | -0.25 | -0.5 |
| BC1a | 0.5 | 0.5 | 0 | 0.25 | -0.5 | 1 | 1 | 0 | 0.25 | 0.25 | 0.25 | 0 | 0 | 0.125 | 0.125 | -0.25 | -0.25 | 0 | 0.5 | 0.5 | 0 | 0.25 | -0.5 |
| BC1b | 0.5 | 0.5 | 0.5 | 0.25 | 0.5 | 1 | 1 | 0 | 0.25 | 0.25 | 0.25 | 0.25 | 0.25 | 0.125 | 0.125 | 0.25 | 0.25 | 0.25 | 0.5 | 0.5 | 0.5 | 0.25 | 0.5 |
| rBC1a | 0.5 | 0.5 | 0.25 | 0.25 | 0.5 | 1 | 0 | 1 | 0.25 | 0.25 | 0.25 | 0.125 | 0.125 | 0.125 | 0.125 | 0.25 | 0.25 | 0.125 | 0.5 | 0.5 | 0.25 | 0.25 | 0.5 |
| rBC1b | 0.5 | 0.5 | 0.25 | 0.25 | 0.5 | -1 | 0 | 1 | 0.25 | 0.25 | 0.25 | 0.125 | 0.125 | 0.125 | 0.125 | 0.25 | 0.25 | 0.125 | -0.5 | -0.5 | -0.25 | -0.25 | -0.5 |
| BC2a | -0.5 | 0.5 | -0.25 | 0.25 | -0.5 | 1 | 0 | 1 | 0.25 | -0.25 | 0.25 | 0.125 | -0.12 | -0.12 | 0.125 | 0.25 | -0.25 | 0.125 | -0.5 | 0.5 | -0.25 | 0.25 | -0.5 |
| BC2b | -0.5 | 0.5 | -0.25 | 0.25 | -0.5 | -1 | 0 | 1 | 0.25 | -0.25 | 0.25 | 0.125 | -0.12 | -0.12 | 0.125 | 0.25 | -0.25 | 0.125 | 0.5 | -0.5 | 0.25 | -0.25 | 0.5 |
| rBC2a | -0.5 | 0.5 | -0.5 | 0.25 | -0.5 | -1 | -1 | 0 | 0.25 | -0.25 | 0.25 | 0.25 | -0.25 | -0.12 | 0.125 | 0.25 | -0.25 | 0.25 | 0.5 | -0.5 | 0.5 | -0.25 | 0.5 |
| rBC2b | -0.5 | 0.5 | 0 | 0.25 | 0.5 | -1 | -1 | 0 | 0.25 | -0.25 | 0.25 | 0 | 0 | -0.12 | 0.125 | -0.25 | 0.25 | 0 | 0.5 | -0.5 | 0 | -0.25 | -0.5 |
| 2BC1b | 0.75 | 0.25 | 1 | 0 | 0.5 | 1 | 1 | 0 | 0.562 | 0.187 | 0.062 | 0.75 | 0.25 | 0 | 0 | 0.375 | 0.125 | 0.5 | 0.75 | 0.25 | 1 | 0 | 0.5 |
| 2BC2a | -0.75 | 0.25 | -0.75 | 0.25 | -0.5 | -1 | -1 | 0 | 0.562 | -0.18 | 0.062 | 0.562 | -0.18 | -0.18 | 0.062 | 0.375 | -0.12 | 0.375 | 0.75 | -0.25 | 0.75 | -0.25 | 0.5 |

**Supplemental Table 3 Consolidation of datasets.** Our synthesis of data is characterized by many cases where we have multiple datasets for the same phenotype and species. To avoid allowing these datasets to dominate downstream analyses we consolidated them. For instance in this table all datasets are crosses among strains of Tribolium castaneum and the phenotype was offspring number. Rather than allowing this to count for 27 datasets these were averaged such that only the mean genetic effects in the final row (bolded) were used in downstream analyses.

| | additive | dominance | epistasis |
|---|---|---|---|
| | 0.2 | 0 | 0.8 |
| | 0 | 0.086 | 0.914 |
| | 0 | 0.502 | 0.498 |
| | 0 | 0.39 | 0.61 |
| | 0.358 | 0.136 | 0.506 |
| | - | - | - |
| | 0.153 | 0.433 | 0.414 |
| | 0 | 0.558 | 0.442 |
| | 0.014 | 0.475 | 0.511 |
| | 0 | 0.382 | 0.618 |
| | 0.014 | 0.421 | 0.565 |
| | 0.264 | 0.146 | 0.59 |
| | 0 | 0 | 1 |
| | 0 | 0.596 | 0.404 |
| | - | - | - |
| | 0.31 | 0 | 0.69 |
| | 0 | 0 | 1 |
| | 0.031 | 0.46 | 0.51 |
| | 0 | 0.453 | 0.547 |
| | 0.032 | 0.421 | 0.547 |
| | 0.332 | 0 | 0.668 |
| | 0 | 0 | 1 |
| | 0.25 | 0.403 | 0.347 |
| | 0 | 0 | 1 |
| | 0.056 | 0.551 | 0.393 |
| | 0 | 1 | 0 |
| | - | - | - |
| mean | **0.084** | **0.309** | **0.607** |

**Supplemental Table 4 Classification of genetic effects.** All genetic effects evaluated in any of the included datasets indicating the way they were pooled into three classes: additive, dominance, and epistatic.

| Class | Possible Genetic Effect |
|---|---|
| Additive | Aa, Xa, Ca, Mea, Za, Wa, Ya |
| Dominance | Ad, Med, Xd |
| Epistatic | AaAa, AaAd, AdAd, AaCa, AdCa, AdXa, XaXa, XaCa, AdYa, XaYa, YaCa, AaXa, AaXd, AaYa, AdXd, XaXd, XdXd, XdYa, XdCa, XaAa, XaAd, YaAa, YaAd, YaXa, CaAd, CaXa, CaYa, CaAa, CaXd, AaZa, AaWa, AdZa, AdWa, ZaZa, ZaWa |

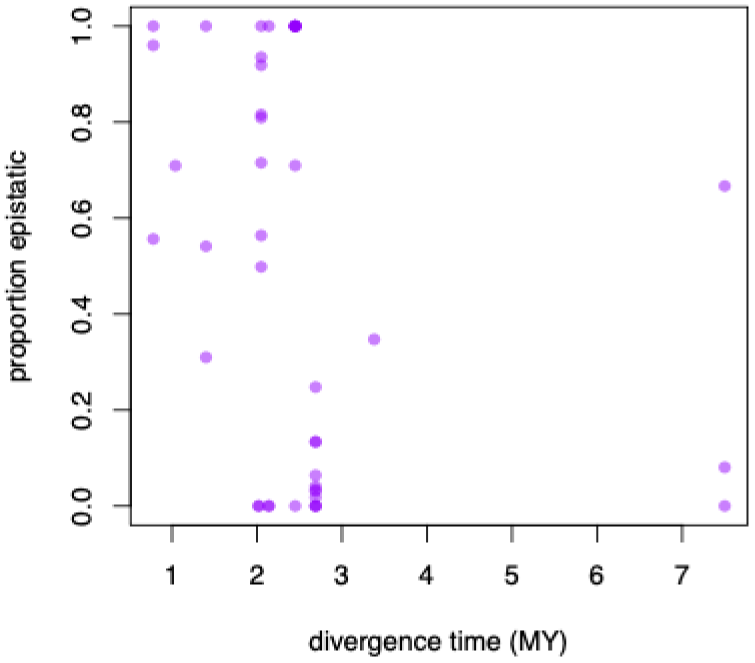


**Supplemental Figure 1 Divergence time and epistatic contribution to trait divergence.** Here we show the relationship between epistatic contribution to trait divergence and divergence time. We did not find a significant correlation between epistatic genetic effects and divergence time (p-value=0.055).
